# Supplementary material for: Use of Patient-Reported Data within the Acute Healthcare Context: A Scoping Review
Source: Int J Environ Res Public Health. 2022 Sep 6;19(18):11160. doi: 10.3390/ijerph191811160 (PMC9517657; doi:10.3390/ijerph191811160)
Supplement: Supplementary file 1 [file ijerph-19-11160-s001.zip › ijerph-1838721-supplementary.pdf]

Supplementary file S1 – search strategy

Search	Query
#1	patient reported outcome*.mp OR treatment intention*.mp OR functional assessment instrument.mp OR functional status questionnaire*.mp OR intention to treat.mp OR (awareness adj3 clinicians).mp OR health personnel attitude.xm OR attitude of health personnel.mp OR patient referral.mp OR physician patient relations.xm OR physician patient relations*.mp OR self report* questionnaire*.mp OR (self administered adj3 questionnaire*).mp OR (self administered adj3 interview).mp OR self report screening.mp OR screening questionnaire*.mp OR self-assessed health status.mp OR self rating scale*.mp OR patient satisfaction.xm OR patient satisfaction.mp OR physician satisfaction.mp OR consumer satisfaction.xm OR consumer satisfaction.mp OR physicians prescription pattern*.mp OR physician attitude.mp OR (recognition adj3 treatment adj3 depression).mp OR (health status report OR health status reports OR health status questionnaire*).mp OR symptom monitor.mp OR cancer related pain.mp OR (communication adj5 patient problem*).mp. OR attention towards symptom*.mp OR (patient satisfaction survey) OR (patient reported experience*) OR (patient reported outcome measure*) OR (prem OR PREM) or (PROMS)
#2	Quality of health care or quality assurance, health care or quality indicators, health care or health plan implementation; patient experience or patient cent* or patient involv* or patient care experience; (Patient or patient experience or patient satisfaction); Quality AND (improve or enhance or raise); Intervention or treatment or training or program*; (quality improv* or quality enhanc* or quality assurance or quality of healthcare)
#3	Primary care.mp OR General practi*.mp OR Primary health*.mp OR Family practice.sh OR family practice.mp OR Family physicians.sh OR family physician*.mp OR Family practice.mp OR Family medicine.mp OR general medical setting*.mp OR medical inpatients.mp OR medical inpatients.mp OR (clinical oncology adj2 practice).mp OR (oncology adj2 clinical practice).mp OR routine oncology practice.mp OR outpatient oncology practice*.mp OR outpatient adj2 oncology clinic*.mp OR cancer outpatient clinic*.mp OR cancer center.mp OR cancer centre.mp OR cancer centres.mp OR oncology department.mp OR (general adj3 medicine clinic).mp OR neurology patient*.mp OR community practice*.mp OR emergency department.mp OR (inpatient OR inpatients).mp OR (palliative phase.mp AND cancer.mp) OR (feedback adj3 oncologist*).mp. OR patient reported cancer need*.mp OR community practice*.mp OR emergency department.mp OR (inpatient OR inpatients).mp ((((((((((hospital) OR (acute care) OR (tertiary care center) OR (tertiary healthcare) OR (emergency department)) OR (emergency room) OR (outpatient clinic)) OR (inpatient) OR (outpatient) OR (ambulatory care) OR ("hospitalized patients")
#4	#1 OR #2
#5	#4 and #3
Limited to January 2009 – June 2020, English, Humans.	
